# Supplementary material for: Single molecule delivery into living cells
Source: Nat Commun. 2024 May 23;15:4403. doi: 10.1038/s41467-024-48608-3 (PMC11116494; doi:10.1038/s41467-024-48608-3)
Supplement: Supplementary file 3 — Description of Additional Supplementary Files [file 41467_2024_48608_MOESM3_ESM.pdf]

Description of Additional Supplementary Files

Supplementary Movie 1: Animation depicting representative translocation simulation from the nanopipette to the electrolyte bath without BSA (example 1)

Supplementary Movie 2: Animation depicting representative translocation simulation from the nanopipette to the electrolyte bath with BSA (example 1)

Supplementary Movie 3: Animation depicting representative translocation simulation from the nanopipette to the electrolyte bath without BSA (example 2)

Supplementary Movie 4: Animation depicting representative translocation simulation from the nanopipette to the electrolyte bath with BSA (example 2)

Supplementary Movie 5: Animation depicting representative translocation simulation from the nanopipette to the electrolyte bath without BSA (example 3)

Supplementary Movie 6: Animation depicting representative translocation simulation from the nanopipette to the electrolyte bath with BSA (example 3)
