# Supplementary material for: Single molecule delivery into living cells
Source: Nat Commun. 2024 May 23;15:4403. doi: 10.1038/s41467-024-48608-3 (PMC11116494; doi:10.1038/s41467-024-48608-3)
Supplement: Supplementary file 10 — Reporting Summary [file 41467_2024_48608_MOESM10_ESM.pdf]

nature portfolio

Corresponding author(s): Paolo Actis, Eric Hewitt

Last updated by author(s): Apr 10, 2014

Reporting Summary

Nature Portfolio wishes to improve the reproducibility of the work that we publish. This form provides structure for consistency and transparency in reporting. For further information on Nature Portfolio policies, see our Editorial Policies and the Editorial Policy Checklist.

Statistics

For all statistical analyses, confirm that the following items are present in the figure legend, table legend, main text, or Methods section.

n/a	Confirmed	
☐	☒	The exact sample size (*n*) for each experimental group/condition, given as a discrete number and unit of measurement
☐	☒	A statement on whether measurements were taken from distinct samples or whether the same sample was measured repeatedly
☒	☐	The statistical test(s) used AND whether they are one- or two-sided<br>*Only common tests should be described solely by name; describe more complex techniques in the Methods section.*
☒	☐	A description of all covariates tested
☒	☐	A description of any assumptions or corrections, such as tests of normality and adjustment for multiple comparisons
☐	☒	A full description of the statistical parameters including central tendency (e.g. means) or other basic estimates (e.g. regression coefficient) AND variation (e.g. standard deviation) or associated estimates of uncertainty (e.g. confidence intervals)
☒	☐	For null hypothesis testing, the test statistic (e.g. *F*, *t*, *r*) with confidence intervals, effect sizes, degrees of freedom and *P* value noted<br>*Give P values as exact values whenever suitable.*
☒	☐	For Bayesian analysis, information on the choice of priors and Markov chain Monte Carlo settings
☒	☐	For hierarchical and complex designs, identification of the appropriate level for tests and full reporting of outcomes
☒	☐	Estimates of effect sizes (e.g. Cohen's *d*, Pearson's *r*), indicating how they were calculated

Our web collection on statistics for biologists contains articles on many of the points above.

Software and code

Policy information about availability of computer code

Data collection	pclamp10,HPICM (Version 1.3.0.11, SICM controller provided by ICAPPIC ltd), Bruker Nanoscope10.00 (AFM image acquisition) and Bruker Nanoscope Analysis (Verion 3.0, for AFM images processing) , ANDOR iQ3 live cell imaging software,ARBD simulation engine (Version Feb22)
Data analysis	Matlab(r2022a) running the Nanopore app provided by Prof Joshua Edel (Imperial College London) version 6.4 , Python (version 3.9)

For manuscripts utilizing custom algorithms or software that are central to the research but not yet described in published literature, software must be made available to editors and reviewers. We strongly encourage code deposition in a community repository (e.g. GitHub). See the Nature Portfolio guidelines for submitting code & software for further information.

Data

Policy information about availability of data

All manuscripts must include a data availability statement. This statement should provide the following information, where applicable:

- Accession codes, unique identifiers, or web links for publicly available datasets
- A description of any restrictions on data availability
- For clinical datasets or third party data, please ensure that the statement adheres to our policy

Data (ionic current traces, fluorescent micrographs, SICM scans) supporting this work can be freely accessed via the University of Leeds data repository

Research involving human participants, their data, or biological material

Policy information about studies with human participants or human data. See also policy information about sex, gender (identity/presentation), and sexual orientation and race, ethnicity and racism.

Reporting on sex and gender	n/a
Reporting on race, ethnicity, or other socially relevant groupings	n/a
Population characteristics	n/a
Recruitment	n/a
Ethics oversight	n/a

Note that full information on the approval of the study protocol must also be provided in the manuscript.

Field-specific reporting

Please select the one below that is the best fit for your research. If you are not sure, read the appropriate sections before making your selection.

[x] Life sciences [ ] Behavioural & social sciences [ ] Ecological, evolutionary & environmental sciences

For a reference copy of the document with all sections, see nature.com/documents/nr-reporting-summary-flat.pdf

Life sciences study design

All studies must disclose on these points even when the disclosure is negative.

Sample size	Stated in the manuscript. The size of the samples was not calculated using power and sample size calculator tools as the measured cellular response was "digital" (either the cell was fluorescent or not after the injection). In any case all experiments were replicated at least 3 times.
Data exclusions	No data were excluded from the analysis
Replication	Data underpinning experimental figures were obtained from at least 3 technical replicates . All attempt of replication were successful .
Randomization	This study was a pilot investigation and randomization was not relevant
Blinding	Data collection and analysis were not blinded as this study was a pilot investigation related to technology development. At this stage of the development, blinding would complicate the experimental setup and workflow leading to inefficiencies in the interpretation of the experimental results.

Reporting for specific materials, systems and methods

We require information from authors about some types of materials, experimental systems and methods used in many studies. Here, indicate whether each material, system or method listed is relevant to your study. If you are not sure if a list item applies to your research, read the appropriate section before selecting a response.

Materials & experimental systems

n/a	Involved in the study	
[x]	[ ]	Antibodies
[ ]	[x]	Eukaryotic cell lines
[x]	[ ]	Palaeontology and archaeology
[ ]	[x]	Animals and other organisms
[x]	[ ]	Clinical data
[x]	[ ]	Dual use research of concern
[x]	[ ]	Plants

Methods

n/a	Involved in the study	
[x]	[ ]	ChIP-seq
[x]	[ ]	Flow cytometry
[x]	[ ]	MRI-based neuroimaging

Eukaryotic cell lines

Policy information about cell lines and Sex and Gender in Research

Cell line source(s)	Hela (CVCL_0030), HUVEC (Promocell, C-12253), Primary Rat Cortical Neurons (A36511, Gibco) from fetal rats. Information about sex was not provided by the supplier.
Authentication	Cell lines were not authenticated and were used as received from the suppliers specified.
Mycoplasma contamination	not tested
Commonly misidentified lines (See ICLAC register)	None of the cell lines used in the study appeared in the Version 12 of the registered misidentified cell lines

Animals and other research organisms

Policy information about studies involving animals; ARRIVE guidelines recommended for reporting animal research, and Sex and Gender in Research

Laboratory animals	Mouse
Wild animals	n/a
Reporting on sex	n/a
Field-collected samples	n/a
Ethics oversight	All animal work carried out at the University of Leeds was approved by the University of Leeds Animal Welfare Ethical Review Body (AWERB) and performed under UK Home Office License P40AD29D7 to Nikita Gamper and in accordance with the regulations of the UK Animals (Scientific Procedures) Act 1986.

Note that full information on the approval of the study protocol must also be provided in the manuscript.

Plants

Seed stocks	*Report on the source of all seed stocks or other plant material used. If applicable, state the seed stock centre and catalogue number. If plant specimens were collected from the field, describe the collection location, date and sampling procedures.*
Novel plant genotypes	*Describe the methods by which all novel plant genotypes were produced. This includes those generated by transgenic approaches, gene editing, chemical/radiation-based mutagenesis and hybridization. For transgenic lines, describe the transformation method, the number of independent lines analyzed and the generation upon which experiments were performed. For gene-edited lines, describe the editor used, the endogenous sequence targeted for editing, the targeting guide RNA sequence (if applicable) and how the editor was applied.*
Authentication	*Describe any authentication procedures for each seed stock used or novel genotype generated. Describe any experiments used to assess the effect of a mutation and, where applicable, how potential secondary effects (e.g. second site T-DNA insertions, mosiacism, off-target gene editing) were examined.*
